# *N*-(2,3-Dimethyl­phen­yl)benzene­sulfonamide

**DOI:** 10.1107/S1600536809002098

**Published:** 2009-01-23

**Authors:** B. Thimme Gowda, Sabine Foro, K. S. Babitha, Hartmut Fuess

**Affiliations:** a Department of Chemistry, Mangalore University, Mangalagangotri 574 199, Mangalore, India; b Institute of Materials Science, Darmstadt University of Technology, Petersenstrasse 23, D-64287 Darmstadt, Germany

## Abstract

In the crystal structure of the title compound, C_14_H_15_NO_2_S, the amino H atom is *trans* to one of the O atoms of the SO_2_ group. Furthermore, the N—H bond is *anti* to the *ortho*- and *meta*-methyl groups of the aromatic ring. The two aromatic rings are tilted relative to each other by 64.8 (1)°. The mol­ecules form zigzag chains along the *a* axis *via* inter­molecular N—H⋯O hydrogen bonds.

## Related literature

For related literature, see: Gelbrich *et al.* (2007 ▶); Gowda *et al.* (2005 ▶); Gowda *et al.* (2008*a* ▶,*b* ▶,*c* ▶); Perlovich *et al.* (2006 ▶).
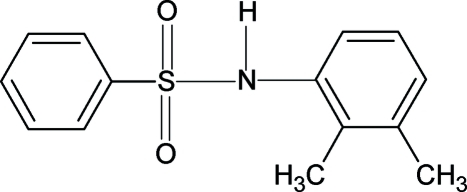


## Experimental

### 

#### Crystal data

C_14_H_15_NO_2_S
*M* *_r_* = 261.33
Orthorhombic, 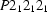

*a* = 6.3969 (5) Å
*b* = 8.8767 (6) Å
*c* = 23.082 (2) Å
*V* = 1310.67 (18) Å^3^
*Z* = 4
Mo *K*α radiation
μ = 0.24 mm^−1^
*T* = 299 (2) K
0.50 × 0.30 × 0.18 mm

#### Data collection

Oxford Diffraction Xcalibur diffractometer with Sapphire CCD detector
Absorption correction: multi-scan (*CrysAlis RED*; Oxford Diffraction, 2007 ▶) *T*_min_ = 0.889, *T*_max_ = 0.958
5869 measured reflections
2611 independent reflections
2200 reflections with *I* > 2σ(*I*)
*R*_int_ = 0.014

#### Refinement

*R*[*F* ^2^ > 2σ(*F* ^2^)] = 0.034
*wR*(*F* ^2^) = 0.094
*S* = 1.07
2611 reflections
168 parameters
H atoms treated by a mixture of independent and constrained refinement
Δρ_max_ = 0.20 e Å^−3^
Δρ_min_ = −0.19 e Å^−3^
Absolute structure: Flack (1983 ▶), 1060 Friedel pairs
Flack parameter: −0.04 (9)

### 

Data collection: *CrysAlis CCD* (Oxford Diffraction, 2004 ▶); cell refinement: *CrysAlis RED* (Oxford Diffraction, 2007 ▶); data reduction: *CrysAlis RED*; program(s) used to solve structure: *SHELXS97* (Sheldrick, 2008 ▶); program(s) used to refine structure: *SHELXL97* (Sheldrick, 2008 ▶); molecular graphics: *PLATON* (Spek, 2003 ▶); software used to prepare material for publication: *SHELXL97*.

## Supplementary Material

Crystal structure: contains datablocks I, global. DOI: 10.1107/S1600536809002098/bt2853sup1.cif

Structure factors: contains datablocks I. DOI: 10.1107/S1600536809002098/bt2853Isup2.hkl

Additional supplementary materials:  crystallographic information; 3D view; checkCIF report

## Figures and Tables

**Table 1:** Hydrogen-bond geometry (Å, °)

*D*—H⋯*A*	*D*—H	H⋯*A*	*D*⋯*A*	*D*—H⋯*A*
N1—H1N⋯O2^i^	0.84 (3)	2.10 (3)	2.936 (2)	176 (2)

Symmetry code: (i) 
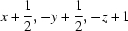
.

**Fig. 1.**
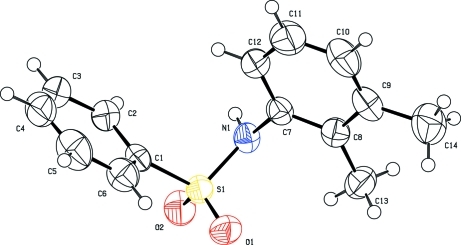
Molecular structure of the title compound, showing the atom labeling scheme. The displacement ellipsoids are drawn at the 50% probability level. The H atoms are represented as small spheres of arbitrary radii.

**Fig. 2.**
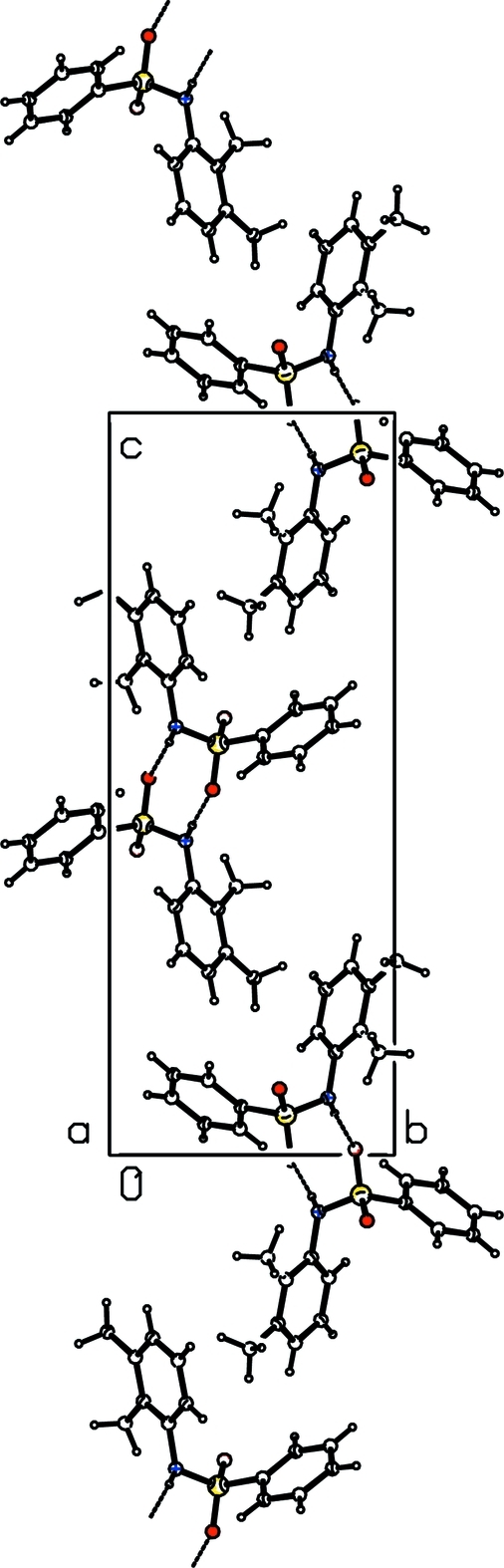
Molecular packing of the title compound with hydrogen bonding shown as dashed lines.

## supplementary crystallographic information

### Comment

In the present work, as part of a study of the substituent effects on the crystal structures of *N*-(aryl)-arylsulfonamides (Gowda *et al.*, 2008*a*, 2008*b*, 2008*c*), the structure of *N*-(2,3-dimethylphenyl)-benzenesulfonamide   has been determined. The amino H atom is trans to one of the O atoms of the SO_2_ group (Fig. 1), similar to that observed in *N*-(2,6-dimethylphenyl)- benzenesulfonamide   (Gowda *et al.*, 2008*a*), *N*-(2-methylphenyl)-benzenesulfonamide (Gowda *et al.*, 2008*b*) and other aryl sulfonamides (Perlovich *et al.*, 2006; Gelbrich *et al.*, 2007; Gowda *et al.*, 2008*c*). The two benzene rings are tilted relative to each other by 64.8 (1)°, compared with the values of 44.9 (1)° in *N*-(2,6-dimethylphenyl)- benzenesulfonamide and 61.5 (1)° in *N*-(2-methylphenyl)-benzenesulfonamide. The other bond parameters of the title compound are similar to those observed in other *N*-(aryl)-sulfonamides. The crystal  packing is stabilized by intermolecular N—H···O hydrogen bonds forming zigzag chains along the a axis (Table 1, Fig. 2).

### Experimental

The solution of benzene (10 cc) in chloroform (40 cc) was treated dropwise with chlorosulfonic acid (25 cc) at 0 ° C. After the initial evolution of hydrogen chloride subsided, the reaction mixture was brought to room temperature and poured into crushed ice in a beaker. The chloroform layer was separated, washed with cold water and allowed to evaporate slowly. The residual benzenesulfonylchloride was treated with 2,3-dimethylaniline in the stoichiometric ratio and boiled for ten minutes. The reaction mixture was then cooled to room temperature and added to ice cold water (100 cc). The resultant solid *N*-(2,3-dimethylphenyl)-benzenesulfonamide was filtered under suction and washed thoroughly with cold water. It was then recrystallized to constant melting point from dilute ethanol. The purity of the compound was checked and characterized by recording its infrared and NMR spectra (Gowda *et al.*, 2005). The single crystals used in X-ray diffraction studies were grown in ethanolic solution by slow evaporation at room temperature.

### Refinement

The C-bound H atoms were positioned with idealized geometry using a riding model with C—H = 0.93 or 0.96 Å. The H atom of the NH group was located in difference map, and its positional parameters were refined freely. All H atoms were refined with isotropic displacement parameters set to 1.2 times of the *U*_eq_ of the parent atom.

To improve considerably values of R1, wR2, and GOOF three  reflections (0 1 1, 0 1 2, 0 1 3) were omitted from the refinement.

### Crystal data

**Table:**

C_14_H_15_NO_2_S	*F*(000) = 552
*M**_r_* = 261.33	*D*_x_ = 1.324 Mg m^−^^3^
Orthorhombic, *P*2_1_2_1_2_1_	Mo *K*α radiation, λ = 0.71073 Å
Hall symbol: P 2ac 2ab	Cell parameters from 2432 reflections
*a* = 6.3969 (5) Å	θ = 2.3–27.7°
*b* = 8.8767 (6) Å	µ = 0.24 mm^−^^1^
*c* = 23.082 (2) Å	*T* = 299 K
*V* = 1310.67 (18) Å^3^	Rod, colourless
*Z* = 4	0.50 × 0.30 × 0.18 mm

### Data collection

**Table:**

Oxford Diffraction Xcalibur diffractometer with Sapphire CCD detector	2611 independent reflections
Radiation source: fine-focus sealed tube	2200 reflections with *I* > 2σ(*I*)
graphite	*R*_int_ = 0.014
Rotation method data acquisition using ω and φ scans	θ_max_ = 26.4°, θ_min_ = 3.3°
Absorption correction: multi-scan (*CrysAlis RED*; Oxford Diffraction, 2007)	*h* = −7→7
*T*_min_ = 0.889, *T*_max_ = 0.958	*k* = −5→11
5869 measured reflections	*l* = −13→28

### Refinement

**Table:**

Refinement on *F*^2^	Secondary atom site location: difference Fourier map
Least-squares matrix: full	Hydrogen site location: inferred from neighbouring sites
*R*[*F*^2^ > 2σ(*F*^2^)] = 0.034	H atoms treated by a mixture of independent and constrained refinement
*wR*(*F*^2^) = 0.094	*w* = 1/[σ^2^(*F*_o_^2^) + (0.0525*P*)^2^ + 0.1566*P*] where *P* = (*F*_o_^2^ + 2*F*_c_^2^)/3
*S* = 1.07	(Δ/σ)_max_ = 0.025
2611 reflections	Δρ_max_ = 0.20 e Å^−^^3^
168 parameters	Δρ_min_ = −0.19 e Å^−^^3^
0 restraints	Absolute structure: Flack (1983), 1060 Friedel pairs
Primary atom site location: structure-invariant direct methods	Flack parameter: −0.04 (9)

### Special details

**Table:**

Experimental. CrysAlis RED (Oxford Diffraction, 2007) Empirical absorption correction using spherical harmonics, implemented in SCALE3 ABSPACK scaling algorithm.
Geometry. All e.s.d.'s (except the e.s.d. in the dihedral angle between two l.s. planes) are estimated using the full covariance matrix. The cell e.s.d.'s are taken into account individually in the estimation of e.s.d.'s in distances, angles and torsion angles; correlations between e.s.d.'s in cell parameters are only used when they are defined by crystal symmetry. An approximate (isotropic) treatment of cell e.s.d.'s is used for estimating e.s.d.'s involving l.s. planes.
Refinement. Refinement of *F*^2^ against ALL reflections. The weighted *R*-factor *wR* and goodness of fit *S* are based on *F*^2^, conventional *R*-factors *R* are based on *F*, with *F* set to zero for negative *F*^2^. The threshold expression of *F*^2^ > σ(*F*^2^) is used only for calculating *R*-factors(gt) *etc*. and is not relevant to the choice of reflections for refinement. *R*-factors based on *F*^2^ are statistically about twice as large as those based on *F*, and *R*- factors based on ALL data will be even larger.

### Fractional atomic coordinates and isotropic or equivalent isotropic displacement parameters (Å^2^)

**Table:**

	*x*	*y*	*z*	*U*_iso_*/*U*_eq_	
C1	0.2823 (3)	−0.0378 (2)	0.43543 (9)	0.0389 (5)	
C2	0.4720 (4)	−0.0422 (3)	0.46456 (11)	0.0507 (6)	
H2	0.5124	0.0374	0.4882	0.061*	
C3	0.5998 (4)	−0.1662 (3)	0.45797 (13)	0.0651 (7)	
H3	0.7262	−0.1708	0.4778	0.078*	
C4	0.5424 (5)	−0.2816 (3)	0.42263 (14)	0.0701 (9)	
H4	0.6285	−0.3655	0.4189	0.084*	
C5	0.3566 (6)	−0.2746 (3)	0.39226 (13)	0.0740 (9)	
H5	0.3206	−0.3519	0.3670	0.089*	
C6	0.2245 (5)	−0.1532 (3)	0.39934 (11)	0.0564 (6)	
H6	0.0974	−0.1496	0.3798	0.068*	
C7	0.2683 (4)	0.2922 (2)	0.36164 (9)	0.0419 (5)	
C8	0.1257 (4)	0.3805 (2)	0.33190 (9)	0.0441 (5)	
C9	0.1664 (4)	0.4088 (3)	0.27267 (10)	0.0536 (6)	
C10	0.3383 (5)	0.3483 (3)	0.24667 (12)	0.0701 (8)	
H10	0.3632	0.3679	0.2077	0.084*	
C11	0.4762 (5)	0.2585 (3)	0.27715 (13)	0.0757 (9)	
H11	0.5909	0.2167	0.2583	0.091*	
C12	0.4452 (4)	0.2308 (3)	0.33475 (11)	0.0566 (7)	
H12	0.5395	0.1724	0.3557	0.068*	
C13	−0.0598 (4)	0.4452 (3)	0.36156 (12)	0.0601 (7)	
H13A	−0.0457	0.5528	0.3637	0.072*	
H13B	−0.0697	0.4045	0.4000	0.072*	
H13C	−0.1837	0.4203	0.3402	0.072*	
C14	0.0210 (6)	0.5071 (4)	0.23845 (14)	0.0840 (10)	
H14A	0.0214	0.6070	0.2544	0.101*	
H14B	−0.1179	0.4664	0.2402	0.101*	
H14C	0.0665	0.5108	0.1988	0.101*	
N1	0.2351 (3)	0.2670 (2)	0.42314 (7)	0.0413 (5)	
H1N	0.337 (4)	0.290 (3)	0.4439 (10)	0.050*	
O1	−0.0648 (3)	0.09633 (19)	0.41129 (7)	0.0567 (5)	
O2	0.0967 (3)	0.13781 (18)	0.50761 (6)	0.0577 (5)	
S1	0.11619 (8)	0.11797 (6)	0.44610 (2)	0.04071 (16)	

### Atomic displacement parameters (Å^2^)

**Table:**

	*U*^11^	*U*^22^	*U*^33^	*U*^12^	*U*^13^	*U*^23^
C1	0.0474 (12)	0.0325 (11)	0.0367 (11)	−0.0028 (9)	0.0072 (10)	0.0039 (9)
C2	0.0512 (14)	0.0446 (13)	0.0562 (15)	0.0015 (11)	−0.0003 (11)	0.0037 (11)
C3	0.0525 (15)	0.0602 (15)	0.0826 (19)	0.0094 (13)	0.0059 (15)	0.0201 (14)
C4	0.074 (2)	0.0485 (16)	0.088 (2)	0.0167 (14)	0.0292 (17)	0.0089 (14)
C5	0.103 (3)	0.0439 (14)	0.0747 (19)	−0.0003 (17)	0.0165 (19)	−0.0139 (13)
C6	0.0650 (16)	0.0453 (14)	0.0588 (15)	−0.0036 (13)	0.0013 (12)	−0.0065 (11)
C7	0.0453 (13)	0.0366 (12)	0.0437 (12)	−0.0046 (10)	−0.0013 (10)	0.0016 (9)
C8	0.0463 (11)	0.0389 (11)	0.0471 (11)	−0.0040 (12)	−0.0060 (10)	−0.0038 (10)
C9	0.0728 (18)	0.0463 (13)	0.0418 (12)	−0.0153 (12)	−0.0039 (12)	0.0044 (10)
C10	0.095 (2)	0.0610 (17)	0.0545 (15)	−0.0060 (16)	0.0127 (16)	0.0077 (13)
C11	0.0747 (19)	0.0694 (19)	0.083 (2)	0.0123 (16)	0.0330 (17)	0.0058 (16)
C12	0.0488 (15)	0.0536 (15)	0.0674 (17)	0.0070 (12)	0.0105 (12)	0.0128 (12)
C13	0.0515 (16)	0.0658 (16)	0.0631 (16)	0.0125 (13)	0.0018 (12)	0.0035 (13)
C14	0.089 (2)	0.094 (2)	0.069 (2)	−0.002 (2)	−0.0176 (18)	0.0251 (17)
N1	0.0473 (11)	0.0380 (10)	0.0384 (10)	−0.0017 (9)	−0.0094 (9)	−0.0001 (8)
O1	0.0425 (10)	0.0555 (11)	0.0721 (11)	−0.0045 (8)	−0.0070 (7)	0.0018 (8)
O2	0.0708 (11)	0.0589 (10)	0.0432 (8)	0.0133 (10)	0.0158 (8)	0.0008 (7)
S1	0.0425 (3)	0.0397 (3)	0.0399 (3)	0.0026 (3)	0.0038 (2)	0.0009 (2)

### Geometric parameters (Å, °)

**Table:**

C1—C6	1.371 (3)	C9—C10	1.363 (4)
C1—C2	1.388 (3)	C9—C14	1.500 (4)
C1—S1	1.762 (2)	C10—C11	1.381 (4)
C2—C3	1.379 (4)	C10—H10	0.9300
C2—H2	0.9300	C11—C12	1.367 (4)
C3—C4	1.360 (4)	C11—H11	0.9300
C3—H3	0.9300	C12—H12	0.9300
C4—C5	1.381 (5)	C13—H13A	0.9600
C4—H4	0.9300	C13—H13B	0.9600
C5—C6	1.380 (4)	C13—H13C	0.9600
C5—H5	0.9300	C14—H14A	0.9600
C6—H6	0.9300	C14—H14B	0.9600
C7—C8	1.385 (3)	C14—H14C	0.9600
C7—C12	1.401 (3)	N1—S1	1.615 (2)
C7—N1	1.453 (3)	N1—H1N	0.84 (3)
C8—C9	1.414 (3)	O1—S1	1.4221 (17)
C8—C13	1.485 (3)	O2—S1	1.4362 (15)
			
C6—C1—C2	120.6 (2)	C11—C10—H10	119.4
C6—C1—S1	120.57 (19)	C12—C11—C10	120.4 (3)
C2—C1—S1	118.80 (16)	C12—C11—H11	119.8
C3—C2—C1	119.2 (2)	C10—C11—H11	119.8
C3—C2—H2	120.4	C11—C12—C7	118.6 (3)
C1—C2—H2	120.4	C11—C12—H12	120.7
C4—C3—C2	120.4 (3)	C7—C12—H12	120.7
C4—C3—H3	119.8	C8—C13—H13A	109.5
C2—C3—H3	119.8	C8—C13—H13B	109.5
C3—C4—C5	120.2 (3)	H13A—C13—H13B	109.5
C3—C4—H4	119.9	C8—C13—H13C	109.5
C5—C4—H4	119.9	H13A—C13—H13C	109.5
C6—C5—C4	120.1 (3)	H13B—C13—H13C	109.5
C6—C5—H5	119.9	C9—C14—H14A	109.5
C4—C5—H5	119.9	C9—C14—H14B	109.5
C1—C6—C5	119.4 (3)	H14A—C14—H14B	109.5
C1—C6—H6	120.3	C9—C14—H14C	109.5
C5—C6—H6	120.3	H14A—C14—H14C	109.5
C8—C7—C12	122.2 (2)	H14B—C14—H14C	109.5
C8—C7—N1	118.4 (2)	C7—N1—S1	121.07 (14)
C12—C7—N1	119.4 (2)	C7—N1—H1N	114.0 (17)
C7—C8—C9	117.3 (2)	S1—N1—H1N	112.4 (17)
C7—C8—C13	121.1 (2)	O1—S1—O2	120.28 (11)
C9—C8—C13	121.6 (2)	O1—S1—N1	107.97 (10)
C10—C9—C8	120.3 (2)	O2—S1—N1	105.37 (10)
C10—C9—C14	119.8 (2)	O1—S1—C1	107.82 (10)
C8—C9—C14	119.9 (2)	O2—S1—C1	106.68 (10)
C9—C10—C11	121.2 (3)	N1—S1—C1	108.24 (9)
C9—C10—H10	119.4		
			
C6—C1—C2—C3	−1.7 (3)	C14—C9—C10—C11	179.4 (3)
S1—C1—C2—C3	177.83 (19)	C9—C10—C11—C12	−1.4 (5)
C1—C2—C3—C4	1.1 (4)	C10—C11—C12—C7	1.6 (4)
C2—C3—C4—C5	1.0 (4)	C8—C7—C12—C11	−0.5 (4)
C3—C4—C5—C6	−2.5 (4)	N1—C7—C12—C11	−178.6 (2)
C2—C1—C6—C5	0.2 (4)	C8—C7—N1—S1	95.3 (2)
S1—C1—C6—C5	−179.3 (2)	C12—C7—N1—S1	−86.4 (2)
C4—C5—C6—C1	1.9 (4)	C7—N1—S1—O1	−45.5 (2)
C12—C7—C8—C9	−0.8 (3)	C7—N1—S1—O2	−175.20 (17)
N1—C7—C8—C9	177.31 (19)	C7—N1—S1—C1	70.97 (19)
C12—C7—C8—C13	−179.5 (2)	C6—C1—S1—O1	−1.1 (2)
N1—C7—C8—C13	−1.4 (3)	C2—C1—S1—O1	179.35 (17)
C7—C8—C9—C10	1.1 (3)	C6—C1—S1—O2	129.3 (2)
C13—C8—C9—C10	179.7 (2)	C2—C1—S1—O2	−50.2 (2)
C7—C8—C9—C14	−178.3 (2)	C6—C1—S1—N1	−117.7 (2)
C13—C8—C9—C14	0.3 (3)	C2—C1—S1—N1	62.79 (19)
C8—C9—C10—C11	0.0 (4)		

### Hydrogen-bond geometry (Å, °)

**Table:**

*D*—H···*A*	*D*—H	H···*A*	*D*···*A*	*D*—H···*A*
N1—H1N···O2^i^	0.84 (3)	2.10 (3)	2.936 (2)	176 (2)

Symmetry codes: (i) *x*+1/2, −*y*+1/2, −*z*+1.
